# Differences in access to coronary care unit among patients with acute myocardial infarction in Rome: old, ill, and poor people hold the burden of inefficiency

**DOI:** 10.1186/1472-6963-4-34

**Published:** 2004-12-09

**Authors:** Carla Ancona, Massimo Arcà, Carlo Saitto, Nera Agabiti, Danilo Fusco, Valeria Tancioni, Carlo A Perucci

**Affiliations:** 1 Department of Epidemiology, Local Health Authority RME, Rome, Italy; 2 Regional Public Health Agency, Friuli Venezia Giulia, Italy; 3 Agency for Public Health, Lazio Region, Italy

## Abstract

**Background:**

Direct admission to Coronary Care Unit (CCU) on hospital arrival can be considered as a good proxy for adequate management in patients with acute myocardial infarction (AMI), as it has been associated with better prognosis. We analyzed a cohort of patients with AMI hospitalized in Rome (Italy) in 1997–2000 to assess the proportion directly admitted to CCU and to investigate the effect of patient characteristics such as gender, age, illness severity on admission, and socio-economic status (SES) on CCU admission practices.

**Methods:**

Using discharge data, we analyzed a cohort of 9127 AMI patients. Illness severity on admission was determined using the Deyo's adaptation of the Charlson's comorbidity index, and each patient was assigned to one to four SES groups (level I referring to the highest SES) defined by a socioeconomic index, derived by the characteristics of the census tract of residence. The effect of gender, age, illness severity and SES, on risk of non-admission to CCU was investigated using a logistic regression model (OR, CI 95%).

**Results:**

Only 53.9% of patients were directly admitted to CCU, and access to optimal care was more frequently offered to younger patients (OR = 0.35; 95%CI = 0.25–0.48 when comparing 85+ to >=50 years), those with less severe illness (OR = 0.48; 95%CI = 0.37–0.61 when comparing Charlson index 3+ to 0) and the socially advantaged (OR = 0.81; 95%CI = 0.66–0.99 when comparing low to high SES).

**Conclusion:**

In Rome, Italy, standard optimal coronary care is underprovided. It seems to be granted preferentially to the better off, even after controversial clinical criteria, such as age and severity of illness, are taken into account.

## Background

The Italian National Health Service is supposed to provide universal coverage of standard care to all citizens, no social or economic selection bias should limit access to high technology resources as far as they are available.

Direct admission to Coronary Care Unit (CCU) on hospital arrival can be considered as a good proxy for adequate management in patients with acute myocardial infarction (AMI) because it has been associated with better prognosis and shorter hospital stay [1,2].

Timely access to advanced diagnostic and therapeutic options, thorough cardiovascular monitoring, provision of primary angioplasty or thrombolytic therapy when indicated, and prompt defibrillation when necessary all contribute to favorable outcomes.

With regard to reperfusion therapies, they have been shown to be effective in reducing short and mid-term mortality in patients with ST-segment elevation AMI [3-8]. The treatment is beneficial regardless of gender, and although relative mortality reduction is greater in younger than in older patients, absolute mortality reduction progressively increases in patients up to 75 years of age. After age 75, the benefits of treatment are less certain [9-11].

Intensive coronary care should be administered without delay, ideally within 60 minutes of the onset of symptoms [12-14].

Previous studies have investigated the role of demographic characteristics (such as age, gender and ethnicity), clinical characteristics (such as time from symptoms, presence of ST elevation and Killip class at presentation), and hospital characteristics (such as location, teaching status and level of invasive capability) on the probability of being admitted to CCU and the probability of receiving initial thrombolysis [11,15-18], but no information is available on factors affecting direct admission to CCU in Italy in an ordinary clinical setting. We analyzed a cohort of patients with AMI hospitalized in Rome (Italy) in 1997–2000 to assess the proportion directly admitted to CCU and to investigate the effect of patient characteristics such as gender, age, illness severity on admission, and socio-economic status on CCU admission practices.

## Methods

### Cohort selection criteria

We used discharge abstract data, routinely collected by the regional Hospital Information System (HIS), to identify a cohort of patients with AMI (ICD-9 code of principal diagnosis at discharge = 410), aged 18 years of age or more, residing in Rome, admitted to one of the 11 city hospitals equipped with an emergency department and a CCU, and surviving the Emergency Room, from 1 July 1997 to 31 December 2000.

From the above defined cohort, we then excluded:

• patients who had been hospitalized for AMI in the previous six months, identified through record linkage within the HIS file,

• patients transferred from other acute care facilities,

• ruled-out AMI (those discharged alive or discharged against medical advice with a length of stay less than five days),

• episodes of care with a diagnosis of trauma, with an important surgical operation, or with DRG non compatible with an AMI diagnosis.

### Exposure and outcome

We categorized patients into five age-intervals: (less than 50, 50–64, 65–74, 75–84 and over 85 years). The Deyo's adaptation of the Charlson's comorbidity index [19] was calculated in order to describe illness severity on admission: based on ICD-9 codes we identified four severity groups (Charlson's adapted index 0, 1, 2, 3+). A socio-economic status (SES) index had been derived for each of the 5736 census tracts (CT) in Rome (average population = 480 inhabitants) using selected census variables including level of education, occupation, dwelling ownership, family size, and people/room density [20]. Based on CT of residence, AMI patients in the cohort were classified into four levels of SES (level I referring to the highest SES).

For each patient, vital status 30 days after hospital admission was obtained through an automatic record linkage with the Municipal Registry of Rome.

The main outcome measure was the indication of CCU as admission ward in the discharge abstract. For the purposes of this study we considered admission to Intensive Care Unit (ICU) in hospitals equipped with CCU equivalent to direct admission to CCU, because both represent an intensive care for AMI patients.

### Statistical analysis

As a first step, a logistic regression analysis was performed in order to confirm the association between direct admission to CCU and 30 days mortality. after adjusting for patient characteristics (gender, age, SES, Charlson's index) and admitting hospital (ORs and 95% CI). Moreover, we compared the average length of stay among patients admitted to CCU and among those admitted to other wards, after excluding deceased and transferred patients, using the Student's *t *test.

We then assessed the extent of differences among Rome hospitals with respect to the number of AMI patients admitted, the size of CCU, and the proportion of AMI patients directly admitted to CCU. For each hospital the ratio between AMI patients admitted and number of CCU beds was calculated as a proxy of the pressure on CCU resources.

The effect of personal characteristics, i.e. gender, age, illness severity and socio-economic status, on risk of non-admission to CCU was then investigated using a logistic regression model. Since important differences among hospital rates of CCU admission had been found, and we wanted to take into account the possible effect of patients clustering by hospital, we used a random effect model with admitting hospital as clustering variable.

The area under the receiver operator characteristics (ROC) curve was estimate as a measure of the overall predictive ability of each model, while the Hosmer and Lemenshow (H-L) statistics was used to assess models' calibration.

The possible effect modification of gender on the other variables was tested by forcing interaction terms in the multivariate models.

All statistical analyses were performed using STATA version 7.0 [21].

## Results

Of 9127 patients hospitalized with an incident AMI diagnosis (32% females, mean age 68.5 years, SD 12.6 years), 53.9% were directly admitted to CCU. The crude 30-day mortality was 13.2% in patients admitted to CCU and 25.0% in those admitted to other wards. The protective effect of CCU admission on 30-day mortality remained strong after adjusting for potential confounders (OR 0.53, I.C. 95% 0.47–0.60; area under the ROC = 0.729; H-L = 7.86, p = 0.448). Moreover, among 3765 AMI patients admitted to CCU or ICU, and discharged to home, the length of stay was significantly shorter than among those admitted to other wards (12.2 versus 14.4 days, p < 0.0001).

The CCU admission proportion varied widely among hospitals (Table 1), from 27.7% in hospital *a *(a large, public, teaching hospital which admitted 962 AMI patients during the study period) to 87.5% in hospitals *l *(a medium sized, no-profit, catholic hospital which admitted 393 AMI patients during the study period). The ratio between AMI patients admitted and number of CCU beds varied from 37 to 166. This two indicators were not slightly correlated (r = 0.52). Patients characteristics with respect to CCU admission status are presented in table 2.

In the bivariate analysis it emerged that lower odds of being directly admitted to CCU were associated to female gender, older age, and higher Charlson's index, while we found no difference with respect to SES level (Table 3, first three columns).

In multiple logistic regression analysis (Table 3, last two columns), when the potential confounding was adjusted for and the clustering by hospital was taken into account, the independent roles of gender (OR = 0.72; 95%CI = 0.64-0.84 when comparing females to males), age (a strong trend over the whole age range, with OR = 0.35; 95%CI = 0.25-0.48 when comparing 85+ to up to 50 yrs.) and illness severity (OR = 0.48; 95%CI = 0.37-0.61 when comparing Charlson index 3+ to 0) were confirmed, while low SES level emerged as an independent determinant of non-admission to CCU (OR = 0.79; 95%CI = 0.65-0.95 and OR = 0.81; 95%CI = 0.66-0.99 when comparing levels III and IV, respectively, to level I, area under the ROC = 0.704; H-L = 15.1, p = 0.06). No effect modification by gender was observed.

## Discussion

Coronary care units have now been in use for 40 years, and it is generally acknowledged that they have helped to improve prognosis and reduce hospital stay among patients with acute myocardial infarction. This was confirmed in our AMI cohort where we observed a strong protective effect of CCU admission on 30 days mortality and a significantly shorter hospital stay for patient admitted to CCU.

We observed that in most Rome hospitals the proportion of AMI patients directly admitted to CCU is lower than it should be according to international recommendations, [12] and lower than that observed in other developed countries [22,23]. Moreover, we found wide differences in rates of CCU admission among hospitals. It is beyond the scope of this paper to investigate which structural and organizational characteristics at the hospital level are associated to high proportion of non-admission to CCU, however admission rates do not increase in hospitals where the number of AMI patients is low in comparison to available CCU beds. While available data, and the results of a previous study [24] suggest a less than optimal use of CCU resources. In fact, we found that, among the 11243 patients who passed through the 112 CCU beds available in the 11 Rome hospitals in the year 2000, for an overall length of stay of 62622 days, only 40% had a diagnosis of AMI, while 46% had principal diagnosis of other acute cardiac disease and 14% had other diagnoses. In summary, variable, and incongruous admission and discharge policies as well as actual shortage of beds could have affected the CCU admission rate of AMI patients, whatever the reasons CCU is apparently a scarce resource in Lazio hospital which should be used unbiasedly.

On the contrary, our results showed that age, severity of illness, and SES are important determinants of the probability that a patient with AMI who reaches qualified Rome hospital is directly admitted to CCU. Previous studies have documented restricted access to CCU and invasive procedures, and under use of well-established therapies such as aspirin, reperfusion and beta-blockers among elderly [25], female [17], and poorer AMI patients [26]. A recent systematic review suggests that patients who are perceived not to benefit from critical care are more often refused intensive care unit admission [27].

The age-related admission policy to CCU we observed has been documented previously [27,28], as well as the lower probability of being accepted in CCU for patients with higher severity [30]. The factors influencing admission decisions are likely to exclude large numbers of patients who could benefit from advanced diagnostic and therapeutic options [31].

We used discharge abstract data, coded according to the International Classification of Diseases IX revision, so it was impossible for us to distinguish between ST-segment elevation and non ST-segment elevation MI. Even though ST-elevation may (and should) influence the physician referral decision, we think that, if the percentages of non-ST segment MI in the groups under study are the same, our results should not strongly be biased.

We used a small area-based SES index, because direct individual data on social class were not available. This index has been shown to be a strong predictor of differences in mortality, [20] and associated to inequalities in access to important health interventions [32,33] and medical management [34] in Rome. Small-area data have been widely used to impute individual socio-economic status, and despite some criticism [35] inferences based on this method appear to be valid [36,37].

Age and admitting hospitals were the variables responsible for a negative confounding effect on the association of socio-economic status with direct CCU admission. Patients with low SES levels are younger than patients with high SES levels and tend to be admitted to hospital with higher provision of CCU care.

## Conclusions

In Rome, where high-technology resources are available, they do not seem to be efficiently and fairly used. Our results suggest that access to optimal care is offered selectively to the socially advantaged, as well as to younger patients (even well under the 75 years threshold) and to those with less severe illness. The National Health Service, its policy of unrestricted access notwithstanding, falls short of providing equal opportunities to all citizens. Delivery of effective services to the underprivileged should be actively promoted.

## Competing interests

The author(s) declare that they have no competing interests.

## Authors' contributions

**CA **conceived of the study, participated in its design, planned the statistical analysis, drafted the manuscript

**MA **participated in the study design and coordination, drafted the manuscript

**CS **participated in the study design, drafted the manuscript

**NA **participated in the study design

**DF **performed the statistical analysis

**VT **assisted in data management

**CAP **conceived of the study

## Pre-publication history

The pre-publication history for this paper can be accessed here:



## Figures and Tables

**Table 1:** Number of CCU beds, AMI patients admitted and directly admitted to CCU, and ratio between AMI admitted and CCU beds by hospital

	**N° of CCU beds**	**AMI patients admitted**	**AMI patients directly admitted to CCU**	**% directly admitted to CCU**	**Ratio between AMI patients admitted and CCU beds**
**Admitting Hospital**					
a	17	962	295	30.7	56.6
b	8	1331	585	43.9	166.4
c	4	639	282	44.1	159.8
d	20	738	361	48.9	36.9
e	9	1414	718	50.8	157.1
f	24	1408	784	55.7	58.7
g	6	678	451	66.5	113.0
h	7	815	552	67.7	116.4
i	7	532	379	71.2	76.0
l	2	217	173	79.7	108.5
m	8	393	344	87.5	49.1
**Total**	**112**	**9127**	**4924**	**53.9**	**81.5**

**Table 2:** Characteristics of AMI patients according to CCU status

	**% not admitted to CCU**	**% directly admitted to CCU**
	(4203)	(4924)
**Gender**		
male	64.26	71.93
female	35.74	28.07
**Age (years)**		
<50	5.14	9.28
50–64	23.65	33.94
65–74	27.84	29.16
75–84	29.31	20.35
85+	14.06	7.27
**Charlson's comorbidity index**		
0	42.76	55.22
1	31.33	30.26
2	15.58	9.38
3+	10.33	5.14
**SES level**		
I	17.74	16.76
II	32.42	31.81
III	28.91	29.85
IV	20.93	21.58

**Table 3:** Effect of patients personal characteristics on risk of non admission to Coronary Care Unit (CCU)

	**Patients**	**% directly admitted to CCU**	**OR**	**95% C.I.**	OR*	**95% C.I.**
**Gender**						
Male	6243	56.7	1.00		1.00	
Female	2884	47.9	0.70	0.64 – 0.77	0.73	0.64 – 0.84
**Age (years)**						
<50	673	67.9	1.00		1.00	
50–64	2665	62.7	0.79	0.67 – 0.95	0.79	0.60 – 1.04
65–74	2606	55.1	0.58	0.48 – 0.69	0.72	0.55 – 0.95
75–84	2234	44.8	0.35	0.32 – 0.46	0.49	0.37 – 0.65
85+	949	37.7	0.29	0.23 – 0.35	0.35	0.25 – 0.48
**Charlson's comorbidity index**						
0	4516	60.2	1.00		1.00	
1	2807	53.1	0.75	0.68 – 0.82	0.69	0.60 – 0.80
2	1117	41.4	0.47	0.41 – 0.53	0.45	0.36 – 0.55
3+	687	36.8	0.38	0.33 – 0.45	0.48	0.37 – 0.61
**SES level**						
I	1523	52.6	1.00		1.00	
II	2840	53.5	1.04	0.91 – 1.17	0.90	0.75 – 1.09
III	2603	54.8	1.09	0.96 – 1.24	0.79	0.65 – 0.95
IV	1883	54.7	1.09	0.95 – 1.25	0.81	0.66 – 0.99

OR: Crude Odds Ratio

OR*: Odds Ratio adjusted for age, gender, severity of illness, and SES. Random effect model with admitting hospital as clustering variable
